# A Case of Balloon Rupture During Coronary Angioplasty: Slow Flow Requiring Swift Action

**DOI:** 10.7759/cureus.9335

**Published:** 2020-07-22

**Authors:** Georgios Sofidis, Anastasios Kartas, Efstratios Karagiannidis, Nikolaos Stalikas, Georgios Sianos

**Affiliations:** 1 Cardiology, American Hellenic Educational Progressive Association University Hospital, Thessaloniki, GRC

**Keywords:** coronary angioplasty, balloon rupture, burst, slow-flow, no-reflow, case report

## Abstract

We describe the case of a middle-aged man undergoing three-vessel coronary angioplasty due to unstable angina. Attempt to predilate a calcified lesion in the left circumflex artery with a semi-compliant balloon, inflated above the rated burst pressure, resulted in balloon rupture. Subsequently, the patient developed ST elevations and became hemodynamically unstable; slow flow in the index vessel was noted. The complication was managed with vasopressor and respiratory support, plus forceful injections of warm saline. Thrombolysis in myocardial infarction (TIMI)-3 flow was eventually restored, and the rest of the procedure was completed uneventfully. Following retrieval of the device, a longitudinal tear in the balloon was observed. This mode of rupture is considered to be safer, when compared to circumferential or pin-hole rupture. Rupture can occur when a balloon is aggressively inflated above nominal pressures and against calcific lesions. The ensuing micro- and macrovascular complications, including slow-flow, no-reflow, vessel dissection or perforation, and intramural hematoma, may induce myocardial ischemia and ultimately cardiogenic shock, malignant arrhythmias, and cardiac arrest. Management should be swift, and relies on supportive measures, depending on the degree of complications caused by the rupture.

## Introduction

Almost every interventional angiographer has or will witness rupture of the angioplasty balloon during a procedure. The energy released from sudden balloon rupture may cause serious arterial injury or compromise coronary flow [1]. Βalloon rupture can be avoided by not exceeding the proposed inflation pressures by the manufacturer, although a good result on angiography frequently depends on high inflation pressures. Our article concerns a case of a balloon inadvertently bursting during a difficult coronary angioplasty procedure, causing hemodynamic collapse to the patient. Given the scant epidemiologic data and the absence of controlled studies to guide decisions on therapy, cumulative experience shared by cases such as this may assist operators performing percutaneous coronary intervention in both preventing and managing this complication.

## Case presentation

A 64-year-old male with known conservatively treated three-vessel coronary artery disease was referred to our catheterization laboratory due to recent aggravation of his angina status despite optimal medical therapy. The SYNTAX (SYNergy Between Percutaneous Coronary Intervention With TAXus and Cardiac Surgery) score was 19. After discussion of the heart team with the patient, percutaneous coronary intervention was decided, aiming to achieve complete revascularization.

**Figure 1:**
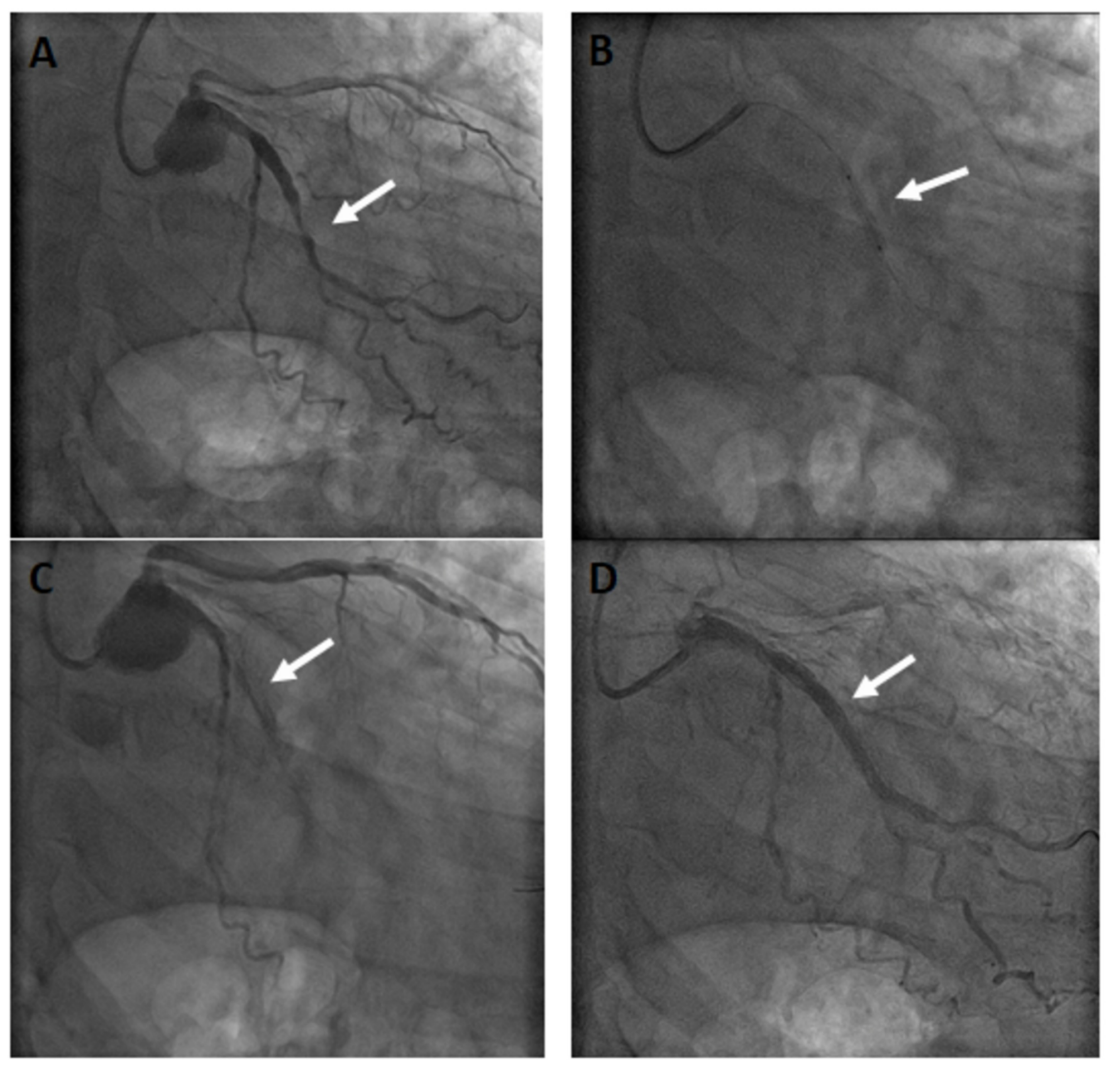
Angiographic images of the LCx angioplasty (Α) A large obtuse marginal branch of the LCx artery featuring a severe, calcified stenotic lesion (arrow). Crossing was successful with a Cougar XT wire (Medtronic, Minneapolis, MN). (B) Positioning and predilation of the lesion of the LCx using a semicompliant balloon. Inflation pressure eventually exceeded the rated burst pressure for the balloon, achieving a sufficient waist yield just before rupture (arrow). (C) TIMI-1 flow after distal embolization of the balloon contents (arrow). (D) Final result. Following restoration of blood flow to the index vessel, two consecutive Promus premier stents (Boston Scientific, Minneapolis, MN) were deployed and a noncompliant Solarice balloon (Medtronic, Minneapolis, MN) was used for postdilatation (arrow). The other two vessels were also treated successfully with angioplasty plus stenting. LCx, left circumflex artery; TIMI, thrombolysis in myocardial infarction.

The procedure started with angioplasty of the left circumflex artery (LCx). An Artimes 2.5 x 20 mm semicompliant balloon (BrosMed Medical, Dongguan, China) was advanced across a calcified target lesion in the middle segment of the vessel (Figure 1A). During progressive inflation to predilate the lesion, the balloon suddenly ruptured at 16 atm, at which point its waist had yielded sufficiently (Figure 1B). The rated burst pressure for that balloon was 14 atm. Instantly, the patient developed severe hypotension with ST elevation in the precordial leads. The balloon was withdrawn and the next contrast infusion revealed thrombolysis in myocardial infarction (TIMI-1) blood flow in the LCx (Figure 1C). The complication was resolved after supportive measures, including oxygen, intravenous noradrenaline infusion, and repeated forceful injection of warm saline. The following contrast infusion showed a good result with TIMI-3 coronary flow, and the rest of the procedure was completed uneventfully (Figure 1D).

**Figure 2:**
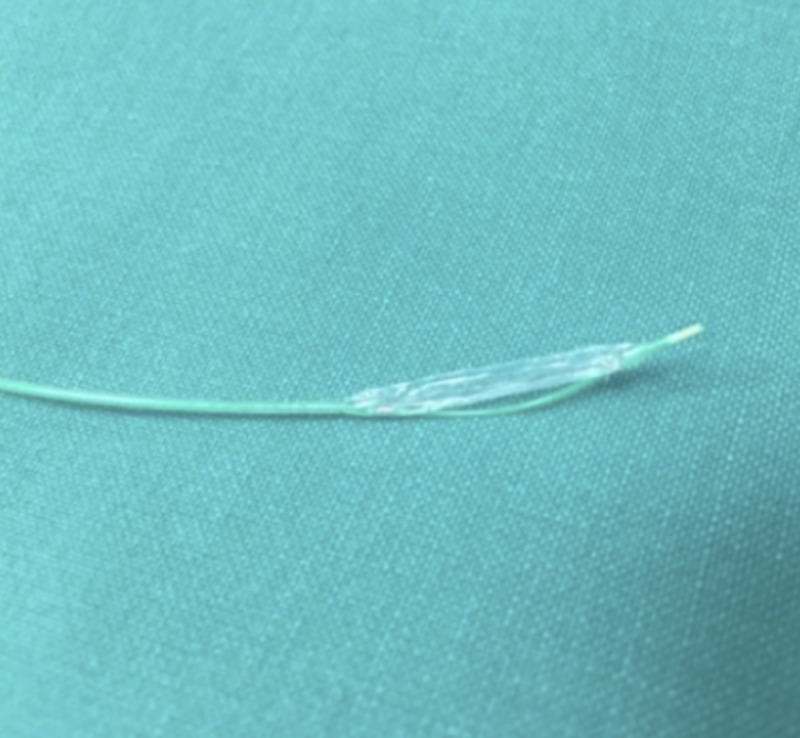
View of the retrieved balloon Inspection of the device, following retrieval. The rupture occurred longitudinally (i.e., along the length of the balloon). In this desired failure mode, the risk of vascular trauma or component detachment is minimized.

On close inspection of the retrieved balloon, a coaxial burst was evident, exposing its inner shaft (Figure 2). The patient was transferred to the coronary care unit, and was discharged the following day in stable condition. 

## Discussion

Balloon rupture is a potentially lethal complication of angioplasty procedures [2,3]. Available data on incidence are outdated [4]. Rupture can occur when a balloon is inflated against a calcified, complex lesion, or when inflation pressure exceeds the rated burst pressure. On fluoroscopy, a quick dispersion of contrast agent and radiolucent bubbles from the balloon may be seen traversing the vessel; inflation pressure is abruptly reduced. Emboli to the distal coronary circulation may cause slow-flow or no-reflow phenomena. The burst, especially when the balloon ruptures in a pin-hole tear, may cause vascular trauma, including local dissection, perforation, and intramural hematoma [5]. A circumferential tear may be associated with detachment of device components [3,6]. By design, and as presented in this case, the balloon tends to rupture in a longitudinal tear, which is less often associated with complications. In total, these micro- and macrovascular complications may induce myocardial ischemia, and ultimately cardiogenic shock, malignant arrhythmias, and cardiac arrest [4,7]. Balloon compliance characteristics should be kept in mind, especially when inflating balloons above nominal pressures and against resistant lesions. This risk for balloon rupture escalates with pressures above the rated burst pressure, until it reaches 50% at average burst pressure. In case of rupture, the operator should withdraw the balloon proximal to the lesion; maneuvering should be careful, so as to avoid entrapment of device materials [8]. Contrast injection should reveal if dissection or perforation is evident, mandating stenting [5]. Management of slow-flow or no-reflow phenomena primarily consists of administering intracoronary vasodilators (e.g. adenosine) while providing aggressive hemodynamic and respiratory support with inotropes/vasopressors, saline, and high flow oxygen, respectively [4,9]. 

## Conclusions

Balloon rupture can be prevented when adhering to the manufacturer’s instructions on inflation pressures. Additional attention on inflation pressures is required when trying to dilate calcific lesions. Ensure that the balloon is purged of all air before inflating the target lesion. A longitudinal tear is considered the most common and safest mode of balloon rupture. The management of balloon rupture is done on a per-case basis, according to the ensuing complications. 
